# Oncological and functional outcomes following treatment of T1a glottic squamous cell carcinoma with transoral laser microsurgery

**DOI:** 10.1186/s40463-021-00553-7

**Published:** 2022-01-20

**Authors:** Dennis E. Curry, David Forner, Matthew H. Rigby, Jonathan R. Trites, Martin Corsten, S. Mark Taylor

**Affiliations:** Division of Otolaryngology – Head and Neck Surgery, Dalhousie University, Suite 3052, Dickson Bldg., 5820 University Avenue, Halifax, NS B3H 1V8 Canada

**Keywords:** Transoral laser microsurgery, Glottic cancer, Oncological outcomes, Voice handicap index-10

## Abstract

**Background:**

Laryngeal cancers of glottic origin comprise a large proportion of head and neck malignancies. Transoral laser microsurgery (TLM) and radiation therapy are mainstays in the treatment of early stage glottic cancer, but debate persists as to which modality is functionally superior. Furthermore, there is a paucity of North American data related to functional and oncological outcomes in T1a glottic cancer. Here, we assessed oncological and functional outcomes of T1a glottic squamous cell carcinoma (SCC) with TLM to supplement evidence from jurisdictions outside North America.

**Methods:**

This study is a retrospective cohort study performed from a prospectively collected tertiary center institutional TLM database. Patients who were diagnosed with T1a glottic SCC and underwent TLM as their primary treatment were included. Functional outcomes were analyzed using the Voice Handicap Index-10 (VHI-10) questionnaire. Ultimate control with TLM only was considered to be those patients with locoregional control with repeat TLM procedures, but without addition of other modalities. Student’s t-test was used to test significance and Kaplan–Meier survival analysis was used to assess oncological outcomes.

**Results:**

48 patients met study criteria. The mean follow-up time was 74 months. The 5-year locoregional, ultimate control with TLM only and laryngeal preservation rates were 83.2%, 90.4% and 100%, respectively. The overall survival and disease-specific survival were 87.2% and 100%, respectively. VHI-10 scores were available for 13/48 patients and mean scores improved non-significantly from pre-op (mean: 11.23; range: 2 to 30; median: 10) and post op (mean: 7.92; range: 0 to 18; median: 8) scoring (p-value = 0.15). Sub-stratification of voice data revealed a significant improvement between pre and post-operative scores (mean difference − 10.6, 95% CI: − 0.99 to − 20.21, p-value = 0.035) for patients with abnormal pre-operative scores (VHI > 11).

**Conclusion:**

To our knowledge, the current work represents one of the first North American studies to report both functional and oncologic outcomes for TLM treatment of T1a glottic SCC. The oncologic and functional outcomes presented here add to existing evidence in favor of TLM as a safe and effective primary treatment option for early staged T1a glottic cancer.

**Graphical abstract:**

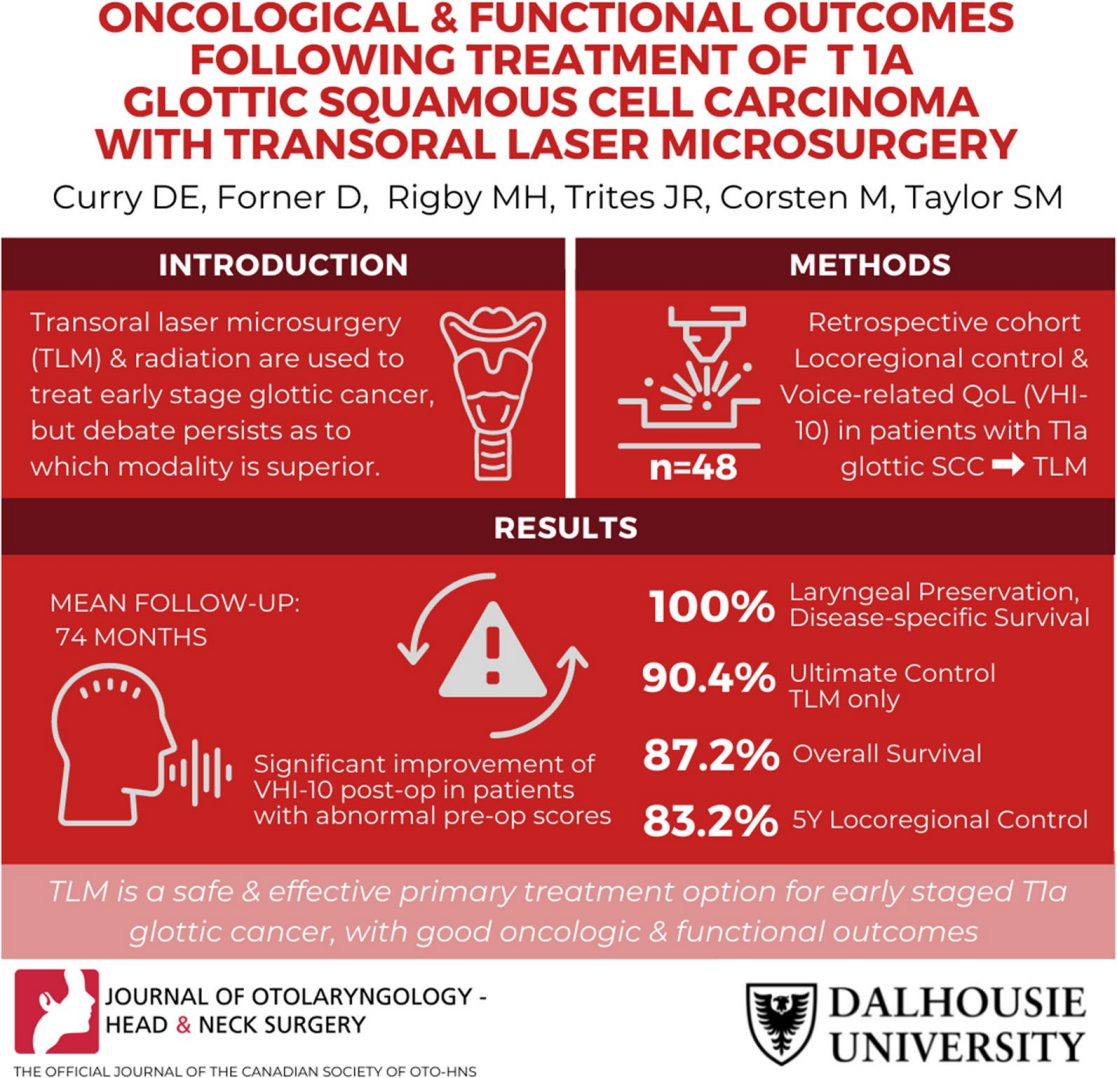

## Background

From its inception as a treatment modality by Strong and Jako [1] to the sentinel work of Steiner [2], transoral laser microsurgery (TLM) has received much attention in the surgical management of head and neck malignancies. Over the past three decades, TLM has been shown to be an effective treatment option for early glottic cancer, with reports also highlighting its utility in late staged cancers [3, 4]. Advantages of TLM over rival treatment options include shorter treatment periods limited to a single surgical day, non-invasive surgical approach leading to rapid recovery times, the ability for repeat procedures, and cost-effectiveness [5]. Still, some debate persists as to whether TLM or radiation therapy (RT) offers superior functional and organ preservation rates [6–10]. Moreover, when it comes to early stage glottic cancer studies from North America in particular, there is a paucity of data detailing both oncologic and functional outcomes for T1a glottic squamous cell carcinoma (SCC) [11, 12]. Large studies that focus on specific stages, such as T1a, provide important evidence for discussions of clinical management and prognosis. It is also paramount to assess the external validity of surgical techniques in diverse patient populations, and outside of originating institutions. We therefore report here on a large single center cohort, demonstrating both functional and oncologic outcomes in the treatment of T1a glottic SCC. With excellent survival, organ preservation, and improved voice outcomes, the current work adds to existing evidence in favor of TLM as an effective treatment option in the management of early stage glottic cancer.

## Methods

### Study design

This study was a retrospective cohort study and relied upon use of our prospectively collected institutional TLM database which has been detailed elsewhere [12]. The project received institutional research ethics board approval prior to the beginning of the study from the Nova Scotia Health Authority Research Ethics Board. Between January 2002 and August 2018, adult patients (≥ 18 years old) diagnosed with cT1aN0M0 squamous cell carcinoma of the glottis were included in the study if they received CO_2_-based TLM as their primary treatment modality. In each case, clinical staging was completed according to the American Joint Committee on Cancer (AJCC) staging criteria and relied on either flexible or indirect laryngoscopy with or without radiological imaging studies. The study followed an intention-to-treat analysis and patients who were upstaged intra- or post-operatively were included in the analysis. Patients who were treated primarily with RT or open surgery before their initial TLM were excluded from the study. Following diagnosis, patients at our center are presented with options of either RT or TLM as a primary modality and choose a treatment course following a discussion with surgical and radiation oncology staff detailing the risks and benefits of each and reflecting on tumor board recommendation and personal preference [13].

### Oncologic and functional outcomes

Oncologic outcomes studied included overall survival (OS), disease-specific survival (DSS), loco-regional control (LRC), ultimate control with TLM only and laryngeal preservation. Ultimate control with TLM only was considered locoregional control after repeated TLM procedures as long as no other form of treatment was utilized such as radiotherapy, alternative laser source, or total laryngectomy. An event in the LRC analysis was defined as either a local or regional recurrence or a second laryngeal primary. An event in the ultimate control with TLM only analysis was defined as RT, Chemo-RT, laryngectomy or refusal of glottic cancer treatment outright. Cases of carcinoma in situ (Cis) were included as recurrences and second primaries were defined as those diagnosed 5 years or more after the original cancer diagnosis. Functional outcomes were assessed using the voice handicap index-10 (VHI-10) laryngeal outcome tool, a 10-part questionnaire that assesses patients’ subjective perception of their voice quality. Extent of initial surgical resection is reported as the European Laryngological Society (ELS) cordectomy classification.

### Statistical analysis

Descriptive statistics were completed for cohort demographics and related patient information as well as voice handicap 10-index (VHI-10) scores. SPSS Statistics® (IBM Corp, Version 24.0. Armonk, NY: IBM Corp.) and Microsoft Excel for Office 365 (Microsoft, Version 1907) were used in data analysis. Post-operative VHI-10 scores were recorded at a minimum of 3 months after the laser procedure and those with both pre and post-operative scores were analyzed. When multiple post-operative scores were documented, the score closest to 12 months post procedure was chosen for analysis. In cases where multiple scores were equivalent in distance from the 12-month mark, the latest score was used in analysis. Student’s t-test was used to test pre- and post-operative VHI-10 scores for significance (alpha = 0.05). Oncologic outcomes were measured using the Kaplan–Meier survival analysis with 5-year rates reported for all metrics.

## Results

In total, 59 patients were identified who underwent TLM for T1a glottic cancer. Following chart and database review, 48 patients met study criteria. Patients were primarily excluded for insufficient documentation to allow outcome review. Demographic information is summarized in Table 1 and shows that the majority of the cohort was male (83%), in keeping with known gender distributions for laryngeal cancer. Smoking status was available for 45/49 patients (90%); 36/45 (80%) had a positive smoking history and 9/45 (20%) were non-smokers. There was a near even distribution between left and right-sided cord involvement and the mean age at treatment was 69 years and ranged between 30 and 87 years. The mean time to last follow-up in the study was 74 months (range: 0–176 months). The majority of primary resections were ELS class II.

**Table 1:** Cohort demographics and ELS classification of resection

Variable	Number (% or range)
*Number of patients*	48
Male	40 (83)
Female	8 (17)
Average age at treatment	69 years (30–87 years)
Average time to last follow-up	74 months (0–176)
*Smoking status available*	45 (94)
Smoking Hx	36 (80)
Non-smoker	9 (20)
Left true cord	22 (46)
Right true cord	26 (54)
*ELS classification*	
Class I	9 (20)
Class II	22 (48)
Class III	11 (24)
Class IV	1 (2)
Class V	3 (7)
Va	1 (2)
Vb	0 (0)
Vc	2 (4)
Vd	0 (0)
Class VI	0 (0)
Missing	2 (4)

### Oncologic outcomes

**Fig. 1:**
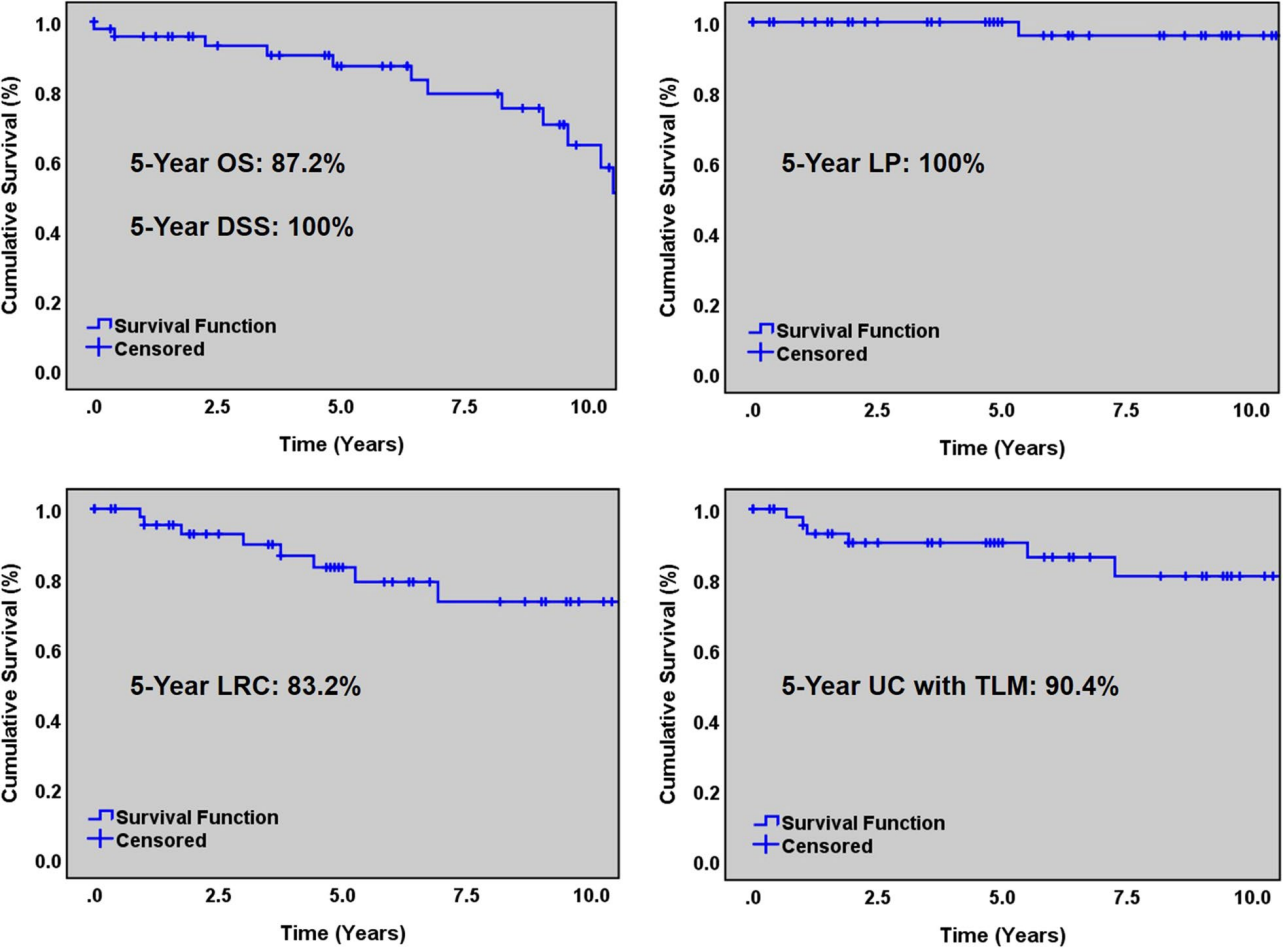
Top: 5-year overall survival (left) and laryngeal preservation (right). Bottom: 5-year locoregional control (left) and ultimate control with TLM only (right)

Five-year rates for overall survival, disease-specific survival, locoregional control, ultimate control with TLM only and laryngeal preservation are shown in Fig. 1. The 5-year OS rate was 87.2%, with five deaths. In total, 14 patients died during the entire study period. All deaths were due to other causes; importantly, DSS was therefore 100%. The 5-year LRC was 83.2%, with 6 patients failing locoregionally. Two additional patients developed second primaries after 5 years. Of the second primaries, one involved both cords and the subglottic area, while the other involved the contralateral cord. The 5-year ultimate control rate with TLM only was 90.4%, representing a 7.2% increase over the 5-year LRC rate. In total 7 patients failed ultimate control with TLM alone, four of which failed within 5 years; 2 received chemoradiation, 4 received radiation and 1 patient refused radiation in favor of potassium titanyl phosphate (KTP) laser therapy at another institution as this was not available at our center at that time.

### Functional outcomes

One patient in the cohort developed post-operative laryngeal bleeding after surgery for a second recurrence, requiring return to the operating room. The patient suffered a cardiac arrest resulting in neurological deficit after return of spontaneous circulation and culminating in tracheostomy and gastrostomy tube requirement.

**Fig. 2:**
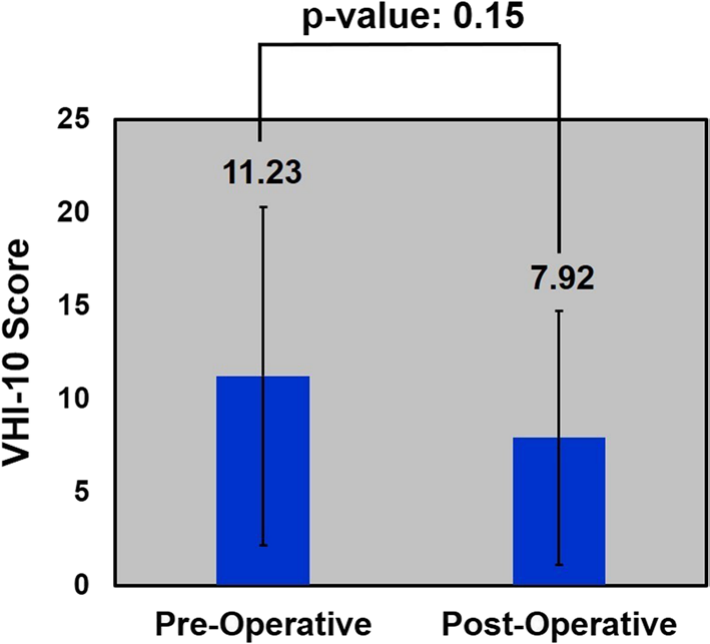
Voice Handicap Index-10 outcomes for patients both pre- and post-operatively. Significance set at p < 0.05

In total, both pre- and post-operative VHI-10 scores were available for 13 of 48 patients studied. Of these patients, a majority had ELS class II resections (n = 5, 38%). The remainder of the VHI subset had ELS class I (n = 4, 31%; class III (n = 3, 23%) and class V (n = 1, 8%). A breakdown of ELS for the entire cohort is included in Table 1. The average pre-operative score was 11.23 (range 2 to 30; median 10) and the average post-operative score was 7.92 (range 0 to 18; median 8), demonstrating a statistically non-significant improvement in functional voice outcomes (p = 0.15) (Fig. 2).

**Fig. 3:**
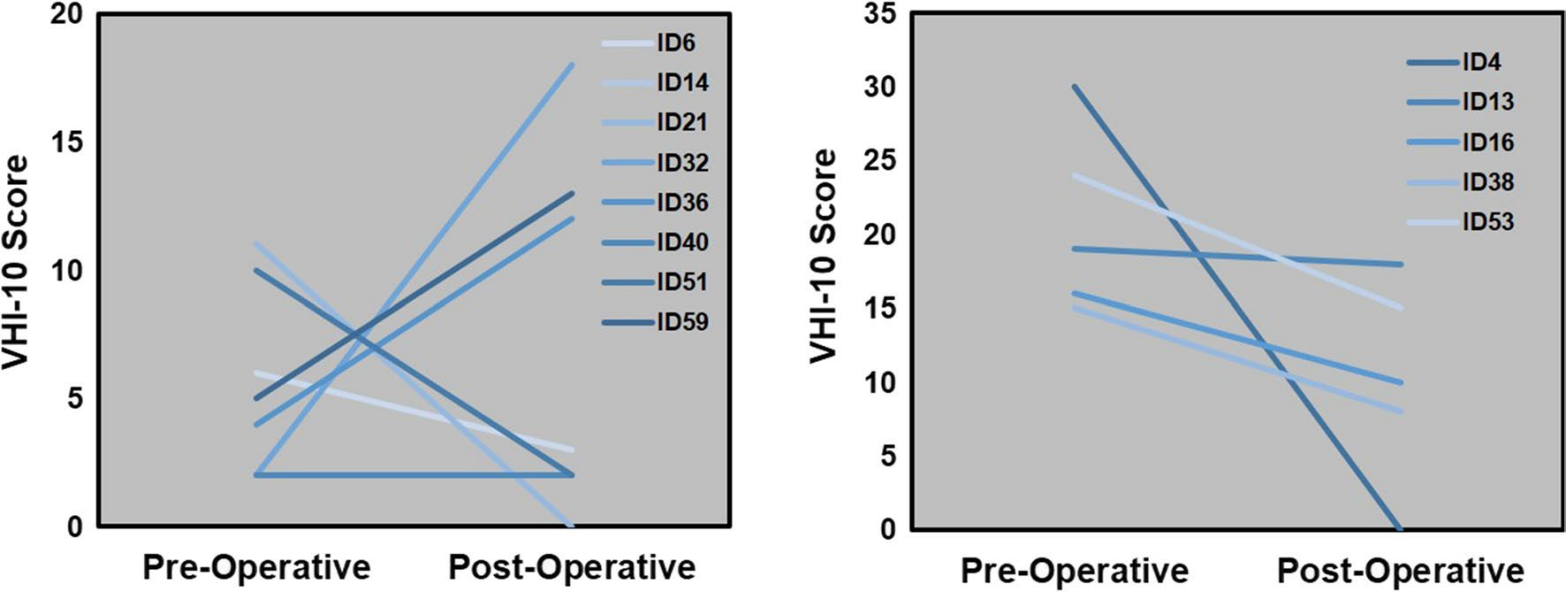
Voice Handicap Index-10 score changes for patients with pre-operative scores of 11 or less (left) and scores above 11 (right)

Given that the average pre-op score for this cohort was close to normal, we sub-stratified patients based on their pre-op scores. We chose a cutoff score of 11 to split the sample into two groups, as scores above 11 have been suggested to be coincide with the perception of abnormal voice quality [14]. Eight patients had pre-op scores of 11 or less and the remaining 5 patients had scores above 11 (Fig. 3). The statistical results are summarized in Table 2 and show a significant improvement between pre- and post-op scores in those patients with clinically abnormal scores (pre-op scores > 11) (p-value: 0.035). No significant difference between pre-operative and post-operative scores was found amongst patients with pre-op scores of 11 or less (p-value: 0.651).

**Table 2:** Comparison of VHI-10 scores for patients considered to have either normal (≤ 11) or abnormal (> 11) pre-operative scores

	Mean pre-op VHI	Mean post-op VHI	Mean difference	95% CI of means	p-value
Pre-op scores ≤ 11 (n = 8)	5.25	6.5	1.25	(− 4.54985)–(7.04985)	0.651
Pre-op scores > 11 (n = 5)	20.8	10.2	− 10.6	(− 0.98642)–(− 20.21358)	0.035

The 5-year laryngeal preservation rate was 100%, and 2 patients required total laryngectomies 5.5 and 12 years following their original TLM treatments. The first of these organ preservation failures received RT following an initial recurrence and total laryngectomy following a second recurrence. The second patient was diagnosed with a T2 second primary, recurred and was treated with RT before receiving total laryngectomy.

## Discussion

Treatment options for early stage glottic cancer include radiation therapy and transoral laser microsurgical approaches with open surgical procedures having become less favored in recent decades [15]. Given its non-invasive nature, cost-effectiveness and excellent outcomes, TLM has gained traction in terms of its adoption, but radiation therapy is still considered the mainstay in many institutions. North American studies reviewing both functional and oncologic outcomes for patients with T1a glottic cancer are scarcely reported. The objective of this study was therefore to highlight TLM functional and oncologic outcomes from a large cohort of T1a patients at a Canadian tertiary care center. Comparison of this study to others is summarized in Table 3.

**Table 3:** Comparison of current work with recent studies investigating oncologic and functional outcomes following TLM for T1a glottic cancer

Study	Year	Study type	Country	TLM cohort size	Stage	OS	DSS	UC with TLM	LP	Voice outcomes
Curry et al. (current work)	2022	Retro	Canada	48	T1a	87	100	90.4	100	VHI-10: significant improvement for those with abnormal pre op scores (> 11)
Canis et al. [17]	2015	Retro	Germany	404	T1a	88	98	NR	97	NR
Schrijvers et al. [23]	2009	Retro	Netherlands	49	T1a	92	100	NR	96	NR
Mahler et al. [24]*	2010	Pro	Norway	188	T1a	88	98	NR	99	NR
Peretti et al. [16]	2010	Retro	Italy	404	T1	NR	99	95	98	NR
Low et al. [8]	2017	Retro	Canada	53	T1a	86	100	100	100	NR
Aaltonen et al. [6]	2014	RCT	Finland	31	T1a	97	97	NR	NR	GRBAS: similar overall function between RT and TLM but voice breathier and glottic gap increased with TLM
Mehel et al. [25]	2019	Retro	Turkey	18	Tis-T1a	NR	NR	NR	NR	VHI: no significant difference between TLM and RT in terms of functional evaluation and overall score; physiological and emotional scores significantly higher in TLM group GRBAS: no significant difference between TLM and RT
Laoufi et al. [26]	2014	Retro	France	44 (voice) 74 (oncologic)	T1a	88	100	NR	NR	VHI: significant difference in favor of RT compared to TLM

For studies including oncologic outcomes, outcomes are measured at 5 years for all studies except Mahler* et al. (2019) which was measured at 3 years and Aaltonen et al. (2014) which was measured at 2 years

*retro* retrospective, *pro* prospective *RCT* randomized control trial, *TLM* transoral laser microsurgery, *OS* overall survival, *DSS* disease-specific survival, *UC* ultimate control, *LP* laryngeal preservation, *RT* radiation therapy, *NR* not reported, *VHI* voice handicap index-10, *GRBAS* grade, roughness, breathiness, asthenia, strain

### Oncologic outcomes

A 2010 analysis of a large cohort of early glottic cancer patients by Peretti et al. reported on various oncologic outcomes [16]. In total, 404 patients were staged as T1 cancers of the glottis (312 T1a and 92 T1b). The 5-year disease specific, locoregional control, and laryngeal preservation rates were 99%, 99.2% and 98.1%, respectively. In 2015, Canis et al. studied 404 patients with T1a glottic cancer treated with CO_2_ laser therapy over a 30-year period in Europe [17]. The 5-year rates for recurrence-free survival, overall survival and disease-specific survival were 76.1%, 87.8% and 98%, respectively. These results are comparable to the results of the present study. Laryngeal perseveration has been identified as an area of contention when it comes to optimal treatment of early stage glottic cancer. Importantly, 5-year laryngeal preservation was found to be high in both large studies cited above and reached 100% in our work. Ultimate control with TLM only was not reported in the Canis study but reached 95% in the Peretti study. In our present work, ultimate control with TLM alone reached 90.4%, demonstrating a roughly 7% increase over the 5-year loco-regional control rate.

A 2018 meta-analysis completed by Guimarães and colleagues comparing transoral laser surgery and RT for the treatment of Tis/T1a glottic cancer demonstrated no statistical differences in overall mortality and local control, a finding consistent with reports elsewhere [10, 18]. The study did, however, find statistically significant differences in DSS and organ preservation favoring laser surgery. A recent systematic review and meta-analysis by Vaculik et al. compared TLM and radiotherapy in patients with T1 glottic cancer [19]. The majority of the 16 studies included provided outcome data stratified for T1a cancers. Transoral laser microsurgery was preferred over RT for OS, DSS and organ preservation; and no differences were found between the modalities with respect to local control. In our work, OS reached 85.7% at 5 years, while both DSS and organ preservation reached 100%.

### Functional outcomes

A 2006 meta-analysis completed by Cohen and colleagues compared RT and TLM and found similar posttreatment VHI scores between the cohorts which included both T1a and T1b patients [20]. The cohorts in both groups largely consisted of T1a patients and so the authors asserted that further study of T1b glottic cancer was necessary. Subsequent studies have shown similar trends suggesting TLM and RT have comparable voice outcomes in the treatment of T1 glottic cancer more generally [21].

The 2018 Guimarães study favored RT when it came to the objective measure of voice [10]. Importantly, the meta-analysis found no significant difference between the treatment modalities in terms of subjective voice scores measured using the VHI tool. VHI has become widely adopted given that it reflects functional success from the perspective of the patient, which, after cure and organ preservation, is amongst the most important metrics associated with patient morbidity.

Randomized controlled trials comparing RT and TLM outcomes in early stage glottic cancer are scarce within the literature. A 2014 report by Aaltonen and colleagues compared 31 male patients treated with laser and 25 male patients treated with radiation and utilized the GRBAS (grade, roughness, breathiness, asthenia, strain) scale to evaluate voice outcomes [6]. Overall voice quality was found to be similar between both groups but TLM was associated with more breathy voice and increased glottic gap compared to RT. The study found also found that less hoarseness-related inconvenience was evident for those treated with RT. Importantly, the follow up period in the study was only 24 months and an assessment of VHI scores was not included in the analysis.

We present both pre and post-operative VHI-10 voice data for nearly 30% of our cohort. VHI-10 scores were shown to non-significantly improve from 11.23 to 7.92 following surgery. VHI-10 scores above 11 have been accepted as abnormal and so while the difference did not reach statistical significance in this study, a positive clinically significant trend is nonetheless identified [14].

Voice data samples were then divided into two groups based on their pre-op scores with 11 being chosen as a cutoff. Recent work proposes that a change in VHI-10 of at least 6 points represents a minimally important difference [22]. In our sample, 4 of 5 patients with preop-scores above 11, representing an abnormal voice, had an improvement of 6 points or more. Conversely only 2 of 8 patients with pre-op scores ≤ 11 had an improvement of 6 points or more. Three patients in this group had an increase in VHI-10 following surgery that was greater than 6 points, representing clinically important worsening of their voice (Fig. 3). Together these results demonstrate a clinically relevant change in VHI-10 amongst patients who had abnormal VHI scores prior to surgery.

## Limitations

Limitations exist in the current study. Common to all cohort studies, whether retrospective or prospective, is susceptibility to confounding and bias. Selection bias may have been introduced by excluding patients with insufficient chart data available for retrospective review. Selection bias may also be apparent with respect to treatment choice on the part of patients with exceptional baseline voice function or those who are professional voice users, and who may therefore choose RT in favor of surgery. As such, those undergoing TLM may have worse voice quality at baseline. Additionally, most patients at our institution do receive TLM for early stage glottic cancer, potentially limiting external validity to specific outside centers. However, this study does offer further evidence similar to that shown by large European groups, demonstrating the overall generalizability of the use of TLM for early glottic cancer [17]. Unfortunately, retrospective cohort studies are limited at times by data availability. Voice outcome data were only available for 13 of the 48 patients in the study, limiting statistical testing between pre-operative and post-operative scores. There is likely an element of Type II error due to this limitation. Sub-stratification of the patients for which voice data was available revealed a significant change in VHI-10 for those with abnormal scores prior to surgery, but the small sample size does limit interpretation. Additionally, VHI-10, while validated, is a subjective representation of voice outcome and the sole metric used in this study and so does have limitations. A potential confounder that could not be controlled for in this study was intensity of smoking history.

## Conclusion

Transoral laser microsurgery has established itself as a primary treatment option in early stage glottic cancer owing to its minimally invasive nature, short treatment duration, ability for repeat laser procedures and low cost. We have demonstrated excellent oncologic outcomes and improved functional outcomes for the treatment of T1a glottic SCC with TLM, offering additional jurisdictional evidence and furthering the overall external validity of the surgical technique.

## Data Availability

The datasets used and/or analysed during the current study are available from the corresponding author on reasonable request.

## Abbreviations

TLM: Transoral laser microsurgery
RT: Radiation therapy
VHI-10: Voice handicap index-10
OS: Overall survival
DSS: Disease specific survival
LRC: Loco-regional control
SCC: Squamous cell carcinoma
AJCC: American joint committee on cancer
Cis: Carcinoma in situ
KTP: Potassium titanyl phosphate
GRBAS: Grade, roughness, breathiness, asthenia, strain
